# How self-disclosure of negative experiences shapes prosociality?

**DOI:** 10.1093/scan/nsae003

**Published:** 2024-01-20

**Authors:** Xiaojun Cheng, Shuqi Wang, Bing Guo, Qiao Wang, Yinying Hu, Yafeng Pan

**Affiliations:** School of Psychology, Shenzhen University, Shenzhen 518060, China; School of Psychology, Shenzhen University, Shenzhen 518060, China; School of Psychology, Shenzhen University, Shenzhen 518060, China; School of Psychology, Shenzhen University, Shenzhen 518060, China; School of Psychology, Shanghai Normal University, Shanghai 200234, China; Department of Psychology and Behavioral Sciences, Zhejiang University, Hangzhou 310058, China; The State Key Lab of Brain-Machine Intelligence, Zhejiang University, Hangzhou 310058, China

**Keywords:** self-disclosure, emotional sharing, prosociality, hyperscanning, fNIRS

## Abstract

People frequently share their negative experiences and feelings with others. Little is known, however, about the social outcomes of sharing negative experiences and the underlying neural mechanisms. We addressed this dearth of knowledge by leveraging functional near-infrared spectroscopy (fNIRS) hyperscanning: while dyad participants took turns to share their own (self-disclosure group) or a stranger’s (non-disclosure group) negative and neutral experiences, their respective brain activity was recorded simultaneously by fNIRS. We observed that sharing negative (relative to neutral) experiences enhanced greater mutual prosociality, emotional empathy and interpersonal neural synchronization (INS) at the left superior frontal cortex in the self-disclosure group compared to the non-disclosure group. Importantly, mediation analyses further revealed that in the self-disclosure (but not non-disclosure) group, the increased emotional empathy and INS elicited by sharing negative experiences relative to sharing neutral experiences promoted the enhanced prosociality through increasing interpersonal liking. These results indicate that self-disclosure of negative experiences can promote prosocial behaviors via social dynamics (defined as social affective and cognitive factors, including empathy and liking) and shared neural responses. Our findings suggest that when people express negative sentiments, they incline to follow up with positive actions.

## Introduction

In everyday life, people frequently share their experiences and feelings, particularly intense emotions, with others. This ‘self-disclosure’ (Rimé *et al*., 2020) elicits a dynamic process that shapes and is shaped by social relationships (Willems *et al*., 2020). Sharing one’s positive emotions with peers can promote the discloser’s subjective well-being (Larsen, 2009; Yilmaz and Arislan, 2013), life satisfaction (Kuppens *et al*., 2008), self-esteem (Ruvalcaba-Romero *et al*., 2017) and interpersonal relationships (Whisman, 2013; Wallace, 2014). Interestingly, sharing happiness was recently found to be closely associated with the level of interpersonal neural synchronization (INS) between discloser and listener (Xie *et al*., 2021). However, the social outcomes of sharing negative experiences remain debated and it is unknown whether INS is involved in the underlying process.

Early studies reported that sharing negative experiences may lead to poor outcomes, for example, expressing thoughts and feelings following the collective trauma of 9/11 predicted negative outcomes (Mark *et al*., 2008). Likewise, for individuals at risk of post-traumatic stress disorder (PTSD), disclosure of negative emotions to those with similar at-risk status was associated with greater levels of PTSD among disclosers (Hoyt *et al*., 2010). Conversely, a recent study showed that individuals could also benefit from sharing daily negative experiences, i.e. frequency online self-disclosure weakens the negative association between negative affect and life satisfaction (Wang *et al*., 2018). According to social penetration theory, self-disclosure process, i.e. the reciprocal sharing of personal information, promotes interpersonal communication developing from shallow to deep (Altman and Taylor, 1973). Previous studies have revealed that everyday social talk can shape the social triad of narrator, audience and social target, with powerful consequences for social structure and group action (Peters and Kashima, 2007). Further, a series of studies provides support for the positive effect of negatively valenced irrelevant information, e.g. irrelevant negative information could enhance positive impressions (Shoham *et al*., 2017). Thus, it is likely that self-disclosure of negative experiences could improve prosociality.

Two paths have been proposed to explain the prosocial effect from self-disclosure of negative experiences. First, empathy may serve as a crucial link between the self-disclosure of negative experiences and the manifestation or inclination of prosocial behavior. When individuals disclose negative emotions, both the discloser and the listener become immersed in a shared negative emotional state. Previous studies have indicated that articulating thoughts and feelings can increase empathy, particularly in the context of relationship conflicts (Sels *et al*., 2021). Additionally, witnessing others in distress or in need of support has been shown to trigger empathy (Crick and Dodge, 1994). Based on the empathy-altruism hypothesis (Batson *et al*., 1991), a heightened state of empathy can enhance individuals’ attention to the needs and feelings of others, thereby fostering prosocial behavior (Coke *et al*., 1978; Batson *et al*., 1995). Second, self-disclosure communication typically follows a symmetrical pattern (Pearce and Sharp, 1973) and often reflects positive feelings such as trust and intimacy (Rubin, 1974). In particular, during emotional sharing, disclosers create a communion with listeners, leading to a temporary feeling of being on the same wavelength—or in sync. Shared states or intentions favored interpersonal liking and social closeness (Rennung and Goritz, 2016; Mogan *et al*., 2017; Xie *et al*., 2021), as well as subsequent mutual prosociality (Hu *et al*., 2017). It was possible that self-disclosure of negative experiences promotes interpersonal liking, thereby facilitating prosocial behaviors. Thus, in the current study, we aimed to examine the role of empathy and interpersonal liking in the prosocial effect of self-disclosure of negative experiences.

Social sharing typically involves two or more individuals, e.g. one discloser sharing experiences with another listener. Given the interactive nature of social sharing, it is imperative to adopt the hyperscanning technique for measuring the brain activity of two or more individuals simultaneously during self-disclosing (Montague *et al*., 2002). Studies using hyperscanning have evidenced the occurrence of INS between two persons engaged in social interactions, such as coordination (Cui *et al*., 2012; Cheng *et al*., 2015) and communication (Jiang *et al*., 2015). INS could reflect the quality of communication and shared understanding across interacting individuals (Friston and Frith, 2015; Schoot *et al*., 2016). Moreover, INS during social interactions was found to predict subsequent cooperation (Li *et al*., 2022) and mutual prosociality (Hu *et al*., 2017). It is unknown, however, whether INS is involved in self-disclosure of negative experiences and, if so, how it relates to social dynamics (e.g. empathy and liking) and prosociality.

This study used participant dyads to test the social outcomes of disclosing negative *vs* neutral experiences of oneself (the self-disclosure group) *vs* a stranger (the non-disclosure control group). During disclosure, both dyad members’ respective brain activity was simultaneously recorded by functional near-infrared spectroscopy (fNIRS) systems. The designated brain regions of interest for our study included the prefrontal cortex (PFC) and the right temporal-parietal junction (rTPJ). These specific areas have demonstrated close associations with mentalization (theory of mind) and the mutual social attention system, both integral to social interaction (Stephens *et al*., 2010; Jiang *et al*., 2015; Hu *et al*., 2017). Notably, the rTPJ is often associated with self-other representation and perspective-taking (Saxe and Kanwisher, 2003), while the PFC plays an important role in empathy and exercises top-down control over cognitive and emotional processes (Kahnt *et al*., 2011; Minati *et al*., 2012; Morelli *et al*., 2014). Moreover, enhanced INS within the PFC and rTPJ has been consistently observed in previous fNIRS-based hyperscanning studies involving interactions between individuals (Cheng *et al*., 2015; Reindl *et al*., 2018). After the disclosure of experiences, participants’ prosociality, empathy and liking toward their partners were measured. The study has two main purposes. First, we examined whether self-disclosure of negative experiences promotes self-evaluated prosociality and how it is related to empathy and liking, as two key social dynamics. Second, we focused on INS during self-disclosure of negative experiences to characterize real-time interactions between discloser and listener from an interpersonal neuroscience perspective (Schilbach *et al*., 2013; Pan *et al*., 2022). Given the close associations among INS, social dynamics and prosociality, we hypothesized that INS plays a mechanistic role in the relationship between self-disclosure of negative experiences and prosociality. Specifically, self-disclosure of negative experiences was thought to promote prosociality through INS.

## Methods

### Participants

We recruited 96 healthy college students (mean age: 20.36 ± 1.72 years) from Shenzhen University. Participants were randomly assigned to 48 same-gender dyads (39 female–female dyads and 9 male–male dyads). Dyad partners had no prior acquaintance with each other. Further, dyads were randomly allocated into two disclosure groups (self-disclosure group or non-disclosure group). The 24 dyads (including 21 female–female dyads) in the self-disclosure group were asked to share their own negative and neutral experiences from a first-person perspective, while the 24 dyads (including 18 female–female dyads) in the non-disclosure group were asked to share negative and neutral experiences of unknown others from a third-person perspective. All participants were right-handed, with normal or corrected-to-normal vision and no history of neurological or psychiatric disorders. Each participant gave written informed consent before taking part in the experiment, and all participants received a reward of ¥ 70–80 to compensate for their time. The study procedure was approved by the Ethical Committee of Shenzhen University (PN-202 200 032). In the current study, no statistical methods were used to predetermine sample size, but our sample size is similar to those reported in previous hyperscanning studies (Takeuchi *et al*., 2017; Bevilacqua *et al*., 2019; Pan *et al*., 2020).

### Experimental materials and measures

#### Neutral and negative experiences

To obtain participants’ real-life, first-hand personal experiences in order to optimally make the experiment naturalistic, the day before the experiment all participants were asked to provide written narratives of two recent personal experiences, one neutral and one negative, each described in 200–400 words. Requesting participants to write down their personal experiences aimed to ensure consistency in the elements of narrative disclosures, thereby minimizing variance introduced by differences in narrative style. Moreover, this approach facilitates the collection of essential disclosure material for the non-disclosure group (narrated experiences about others).

The neutral experiences reflected participants’ typical day, such as, ‘In the morning, I went to the Social Science Methodology class at the Arts Building, and it wrapped up around 10:15 am…’ For negative experiences, participants were asked to recall events that caused extensive stress and negative emotions, such as anger, pain and sadness. Descriptions of these events needed to include details like the place, date, time and other relevant facts (e.g. background, unfolding of the event and ending). Here is an excerpt from a negative experience: ‘Last Tuesday, after enjoying a meal of rice noodle rolls at the local eatery, I returned to my dormitory for a midday nap. Unexpectedly, I was struck by a sudden bout of stomach illness, feeling extremely uncomfortable. Over the next two days of illness, I experienced daily nausea, confined to my bed with little ability to do anything.’

Finally, each participant evaluated the valence of their described events by completing the Chinese Mood Adjective Checklist (CMACL; Zhong and Qian, 2005). The CMACL has 16 items across four dimensions: ‘Fidget’ (*F*), ‘Happy and Excited’ (*HE*), ‘Pain and Sad’ (*PS*) and ‘Anger and Hate’ (*AH*). Our study mainly focuses on the three dimensions related to negative emotions (*F, PS* and *AH*). Participants’ ratings on these dimensions were averaged to give a score reflecting the degree of negative emotions. As intended, the negative experiences used in our study scored higher for negative emotions than the neutral experiences (*t* (47) = 10.91, *P *< 0.001, Cohen’s *d* = 1.57, 95% confidence interval (CI) = [1.10, 1.59]). In the experiment, every written narrative would be provided to two participants (i.e. the participant who experienced it in the self-disclosure group and a random participant in the non-disclosure group) as the outline of experience they needed to share. Specifically, the first-person pronoun ‘I’ in the written narrative for the non-disclosure group was replaced with the third-person pronoun ‘he/she’.

#### Prosociality

Participants’ prosociality toward their partners was evaluated using materials adapted from previous studies (Oswald, 2002; Hu *et al*., 2017). Specifically, participants read the following text:

One afternoon, you’re on your way to the cinema to see a long-awaited movie. At the same time, you happen to see the partner. Your partner tells you that she/he can’t find their way to the classroom. She/He is worried and anxious, but the classroom is far away from the cinema and the route is complicated. If you decide to take her/him to the classroom, you will miss the movie.

Participants were asked how much time they would be willing to spend helping their partner. They needed to state a time in whole minutes from 0 to 50.

#### Empathy

We adapted Koehne *et al*.’s (2016) four-item subjective measure to evaluate participants’ empathy for their partners immediately after sharing (Koehne *et al*., 2016). Two items measure cognitive empathy (i.e. ‘To what extent can you understand your partner’s feelings in the story?’ and ‘When listening to your partner’s narrative, can you imagine yourself as the protagonist in the story to understand their feelings and thoughts?’). The other two items measure emotional empathy (i.e. ‘Would seeing your partner happy make you happy too?’ and ‘Would seeing your partner sad make you sad too?’). Participants responded to each item on a seven-point Likert scale (1 = ‘not at all’, 7 = ‘very much’). Emotional empathy and cognitive empathy were separately analyzed.

#### Liking

We adapted two items from Shin *et al*. (2019) to evaluate participants’ liking toward their partners: ‘After this short meeting, how much did you like your partner?’ and ‘If possible, to what extent were you willing to have in-depth communication with your partner (in a potential subsequent encounter)?’ Participants responded to each item on seven-point Likert scales (first item: 1 = strongly dislike, 7 = strongly like; second item: 1 = very unwilling, 7 = very willing).

### Experimental tasks and procedures

**Fig. 1.**
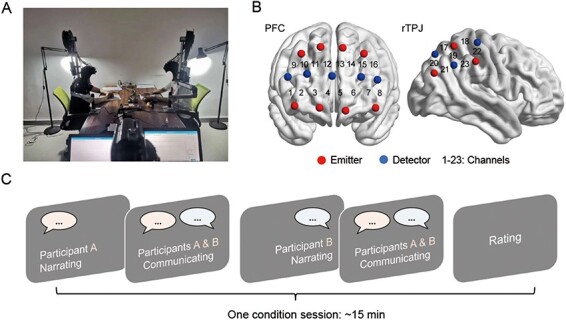
Experimental design. (A) Experimental setup. (B) Probe configuration to measure brain activity: 16 channels on the PFC and 7 channels on the rTPJ. (C) Time flow of one condition session.

In the experiment, the two dyad members (A and B) were seated at a table facing each other at a distance of ∼90 cm, in a silent laboratory with dim fluorescent lights (Figure 1A). They were instructed to spend the first 3 min relaxed, quiet and as motionless as possible, adjusting to the conditions they were in and being ready for the experiment. Next, they briefly introduced themselves to their partners, giving their names and their university majors. This practice was to help participants soothe the awkwardness of the first meeting.

Subsequently, participants engaged in two valence conditions in sequence, one for sharing neutral experiences (the neutral condition) and one for sharing negative experiences (the negative condition). Dyads were required to begin by completing the neutral condition before proceeding to the negative condition. In the current study, dyads underwent identical procedures, regardless of the type of disclosure. The sole distinction between the groups was that dyads in the self-disclosure group shared their personal experiences, while dyads in the non-disclosure group shared experiences belonging to a third, unrelated individual (provided by the experimenter).

In each condition session, participants A and B took turns sharing experiences (Figure 1C). The order alternated between the two valence conditions. A complete sharing process for one participant included a ‘narration’ phase and a ‘communication’ phase. In the narration phase, participant A (or B) narrated the experience to participant B (or A), who just listened without giving any verbal feedback (physical feedback in terms of e.g. facial expressions was not explicitly forbidden). When participant A (or B) finished narrating, participants A and B freely communicated with each other about the experience. The two-phase sharing process lasted ∼7–8 min. When both participants A and B had completed sharing their experiences (after about 15 min), the task session ended. Participants then immediately completed the subjective measures of prosocial behavior, empathy and interpersonal liking toward their partner. They also evaluated the valence of the experience their partner had shared by filling out the CMACL. When completing these measures, they could not and were not allowed to see each other’s responses to the questions.

### fNIRS data acquisition

We used two identical portable fNIRS systems (NirScan Inc., HuiChuang, China) to collect brain activity from both dyad members simultaneously during the experiment. For each participant, two sets of optode probes covered two respective brain regions: the PFC was monitored with 8 emitters and 5 detectors, constituting 16 channels (denoted by ‘CH’ and a number below); the rTPJ was monitored with 3 emitters and 3 detectors, forming 7 channels (Figure 1B). The references for the two probes were FPz (for PFC) and C6 (for rTPJ), in accordance with the international 10/20 system. The distance between the adjacent emitters and detectors was ∼3 cm, and the absorption of near-infrared light (wavelengths: 760 and 850 nm) was measured at a sampling rate of 10 Hz.

### Data analysis

#### Subjective measurements

To verify that there was no disclosure group difference concerning perceived valence of narratives of neutral and negative experiences, we conducted repeated-measures analysis of variance (ANOVA) on participants’ ratings (*N* = 96) of experiences shared by their partner during the experiment. The disclosure group (self-disclosure *vs* non-disclosure) was the between-dyad variable and the valence condition (neutral *vs* negative) was the within-dyad variable.

We also conducted repeated-measures ANOVAs (*N* = 96) for the individual measures of prosociality, cognitive empathy, emotional empathy and liking, again using disclosure group (self-disclosure *vs* non-disclosure) was the between-dyad variable and the valence condition (neutral *vs* negative) as the within-dyad variable. Further, we used Pearson correlation analyses to explore relationships between the subjective measures.

#### fNIRS data

In our study, the light intensity data were automatically converted into hemoglobin concentration [i.e. the oxy-hemoglobin (HbO) and deoxy-hemoglobin (HbR) signals] based on the modified Beer–Lambert Law, and the HbO and HbR signals were directly exported from the fNIRS systems using built-in functions. Our study mainly focused on HbO signals given their higher sensitivity to changes in regional cerebral flow (Hoshi, 2003; Hillman, 2014). For data preprocessing and calculation of INS, we used custom codes in MATLAB software (2022b, MathWorks). During preprocessing, we first used correlation-based signal improvement (CBSI) to reduce head motion artifacts (Cui *et al*., 2010; Reyes *et al*., 2018). CBSI was based on the principle that the HbO and HbR signals should be negatively correlated. Its function is defined as:


$${x_0}\; = \frac{1}{2}\,\,\left( {x-{\ }\alpha y} \right)\\[-5pt]$$

$${y_0}\,\; = \; - \,\frac{1}{\alpha }{x_0}$$

where *x, y, x*_0_ and *y*_0_ represent raw HbO, raw HbR, corrected HbO and corrected HbR signals, respectively, and *α* is the ratio of the standard deviation of raw HbO and raw HbR signals. Specifically, the correlation between *x*_0_ and *y*_0_ should be maximally close to −1. The CBSI method not only removed large-amplitude spikes but also reduced non-spike noise, enhancing the contrast-to-noise ratio of the signal quality (Cui *et al*., 2010). Next, we employed a wavelet-based denoising method to remove global physiological noise (Duan *et al*., 2018). According to this method, the time–frequency points in hemoglobin dynamics affected by global physiological noise were identified; subsequently, the fNIRS signal was decomposed using the wavelet transform and the wavelet power at the identified contaminated time–frequency points was suppressed. This method effectively removed the global physiological noise from the fNIRS signal and improved the spatial specificity of the task activation (Duan *et al*., 2018). Both methods align with the principles outlined in recent recommendations for fNIRS data processing (Yücel *et al*., 2021). They are designed for noise reduction after conversion to hemoglobin due to their rationales and have been successfully used in recent fNIRS hyperscanning studies (Pan *et al*., 2023).

After data preprocessing, we used the wavelet transform coherence approach (Grinsted *et al*., 2004) to estimate the INS between a discloser and a listener for each valence condition and each dyad (48 dyads in total). We averaged across time the INS of each valence condition (neutral *vs* negative) and converted into Fisher-*z* statistics to obtain two 2D matrixes (frequency × channel). We mainly focused on the frequency band of 0.01–0.5 Hz (2–100 s) to avoid INS caused by physiological signals such as heartbeat (∼1 Hz; Guijt *et al*., 2007).

Within this frequency band, we employed a data-driven approach to identify more specific frequencies and channels related to the task. Specifically, we conducted repeated-measures ANOVAs on the INS for each channel and frequency, with valence condition as the within-dyad variable and disclosure group as the between-dyad variable. The analysis yielded three (i.e. two for main effects and one for interaction effect) *P*-value matrixes (68 frequency × 23 channel). The false discovery rate method was used for the three matrixes, respectively, to correct for multiple testing (Benjamini and Hochberg, 1995). This procedure identified one channel (CH1: frequency 0.028–0.036 Hz) demonstrating a significant main effect of valence condition and two channels (CH1: frequency 0.25–0.34 Hz; CH6: frequency 0.09–0.10 Hz) demonstrating a significant interaction effect of valence condition and disclosure group. Importantly, the observed frequency bands (∼0.09 to 0.3 Hz) were consistent with the findings from previous studies on INS in the context of communication (Wang *et al*., 2022a,b). The frequency (0.09–0.3 Hz = 3.3–11.1 s) also roughly corresponds to the average turn-taking time in communication tasks (Balters *et al*., 2023). Therefore, we believed that the INS within these frequency bands, which formed our frequencies of interest, was relevant to our task manipulation. The INS results yielded a *t*-map covering the frontal area. The *t*-map was generated with a spatial interpolation method and rendered over a standard brain template (ICBM 152 nonlinear asymmetric template) using the ‘EasyTopo’ toolbox (Tian *et al*., 2013). For CH1 and CH6, we further used Pearson correlation analyses to explore relationships between INS and the subjective measures. Note that for the correlation analyses, subjective measures of the two dyad members were averaged.

## Results

### Validating the valence of negative and neutral experiences

We conducted repeated-measures ANOVAs on participants’ evaluation of the valence of experiences shared by their partner. Only the main effect of valence condition was found, with participants perceiving a greater degree of negative emotions in the negative condition than in the neutral condition (*F* (1, 94) = 91.41, *P *< 0.001, *η*^2^_partial_ = 0.49, 95% CI = [0.63, 0.96]). This finding indicated that our manipulation was valid.

### Prosociality, empathy and liking

**Fig. 2.**
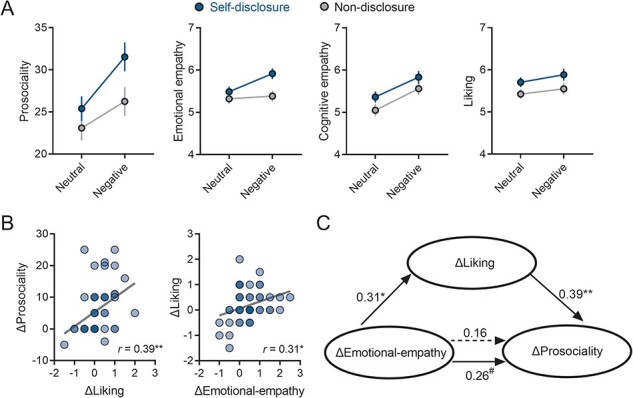
Results for subjective measures. (A) Self-reported prosociality, emotional empathy, cognitive empathy and liking in each disclosure group and each valence condition. (B) The relationships among ΔProsociality, ΔLiking and ΔEmotional-empathy in the self-disclosure group. (C) The mediating role of ΔLiking between ΔEmotional-empathy and ΔProsociality in the self-disclosure group. All estimates were standardized. *P < 0.05, ^**^P < 0.01, ^#^*P* < 0.1.

Valence condition had main effects on self-reported prosociality (*F* (1, 94) = 42.09, *P* < 0.001, *η*^2^_partial_ = 0.31, 95% CI = [3.22, 6.07]), emotional empathy (*F* (1, 94) = 12.07, *P* < 0.001, *η*^2^_partial_ = 0.11, 95% CI = [0.11, 0.39]), cognitive empathy (*F* (1, 94) = 15.97, *P* < 0.001, *η*^2^_partial_ = 0.15, 95% CI = [0.25, 0.73]) and liking (*F* (1, 94) = 6.15, *P* = 0.02, *η*^2^_partial_ = 0.06, 95% CI = [0.03, 0.27]) (Figure 2A). These results indicate that the scores for all four subjective measures were higher after sharing negative experiences than after sharing neutral experiences. Disclosure group had a main effect only on emotional empathy (*F* (1, 94) = 4.72, *P* = 0.03, *η*^2^_partial_ = 0.05, 95% CI = [0.03, 0.67]), which was higher in the self-disclosure group than in the non-disclosure group. More importantly, we observed a significant interaction effect of disclosure group and valence condition on prosociality (*F* (1, 94) = 4.39, *P* = 0.04, *η*^2^_partial_ = 0.05, 95% CI = [0.48, 10.10]) and emotional empathy (*F* (1, 94) = 6.69, *P* = 0.01, *η*^2^_partial_ = 0.06, 95% CI = [0.18, 0.89]). Further analyses revealed that the effects of valence condition on prosociality (*t* (94) = 2.10, *P* = 0.04, 95% CI = [0.16, 5.84]) and emotional empathy (*t* (94) = 2.59, *P* = 0.01, 95% CI = [0.08, 0.64]) were significantly greater in the self-disclosure group (*vs* non-disclosure group) (Figure 2A).

To explicate the interaction effect results and to further examine the valence condition effect (negative *vs* neutral) in separate groups, we focused on the difference in prosociality values between the two valence conditions (ΔProsociality, denoting the negative condition value minus the neutral condition) and its associations with the respective differences in emotional empathy values (ΔEmotional-empathy) and Liking values (ΔLiking). Including such an active condition (specifically, the neutral condition) as a baseline has been demonstrated to enhance the statistical robustness and rigor of the findings (Stark and Squire, 2001; Reindl *et al*., 2018; Hu *et al*., 2022).

Pearson correlation analyses demonstrated that in the self-disclosure group, ΔProsociality was significantly correlated with ΔLiking (*r* = 0.39, *P* = 0.01), which was itself related to ΔEmotional-empathy (*r* = 0.31, *P* = 0.03; Figure 2B). Additionally, ΔProsociality was also marginally correlated with ΔEmotional-empathy (*r* = 0.26, *P* = 0.07). To further explore the relationship among ΔEmotional-empathy, ΔLiking and ΔProsociality, we conducted a mediation analysis for the self-disclosure group with ΔEmotional-empathy, ΔLiking and ΔProsociality as the independent, mediating and dependent variables, respectively. This analysis revealed a significant mediation effect (bootstrap ab = 1.09, 95% CI = [0.06, 2.97]; Figure 2C), suggesting that ΔLiking mediates the relationship between ΔEmotional-empathy and ΔProsociality.

In the non-disclosure group, we again found that ΔProsociality was significantly correlated with ΔLiking (*r* = 0.36, *P* = 0.01) and ΔEmotional-empathy (*r* = 0.34, *P* = 0.02) and that ΔLiking was associated with ΔEmotional-empathy (*r* = 0.48, *P* = 0.001). However, in the mediation analysis with ΔEmotional-empathy, ΔLiking and ΔProsociality as the independent, mediating and dependent variables, respectively, the mediation effect was not significant (bootstrap ab = 1.24, 95% CI = [−0.16, 2.77]).

In brief, enhanced prosociality, emotional empathy, cognitive empathy and liking were found after mutually sharing negative experiences (*vs* sharing neutral experiences). The effects of valence condition (neutral *vs* negative) on prosociality and emotional empathy were predominantly greater in the self-disclosure (*vs* non-disclosure) group. Specifically, in the self-disclosure group, emotional empathy affected prosociality through liking.

### Interpersonal neural synchronization (INS)

**Fig. 3.**
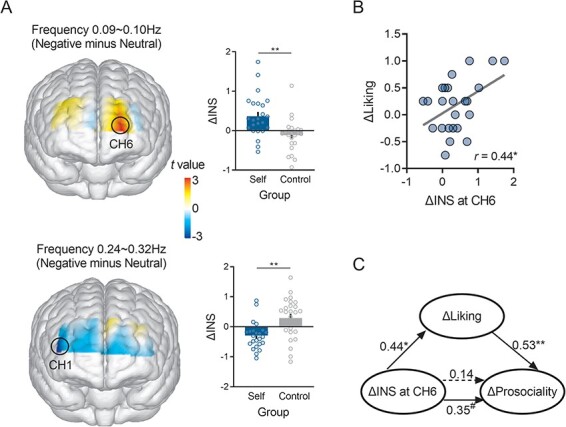
INS analysis results. (A) The effect of disclosure group on ΔINS. (B) The association between ΔINS at CH6 and ΔLiking in the self-disclosure group. (C) The mediation of ΔLiking between ΔINS at CH6 and ΔProsociality in the self-disclosure group. All estimates were standardized. *P < 0.05, ^**^P < 0.01, ^#^*P* < 0.1.

We conducted repeated-measures ANOVAs on INS for each channel and frequency, with disclosure group (self-disclosure *vs* non-disclosure) as the between-dyad variable and valence condition (neutral *vs* negative) as the within-dyad variable. The results revealed that INS at CH1 (frequency 0.028–0.036 Hz) demonstrated significant main effect of condition (*F* (1, 46) = 11.95, *P* = 0.001, *η*^2^_partial_ = 0.21, 95% CI = [0.12, 0.44]), with larger INS in the negative condition compared to that in the neutral condition. More importantly, we found interaction effects of valence condition and disclosure group on INS: first, in the frequency 0.09–0.10 Hz, we found a significant interaction at CH6 (*F* (1, 46) = 12.15, *P* < 0.001, *η*^2^_partial_ = 0.21, 95% CI = [0.17, 0.56]). This interaction remained significant when we added gender into the statistical model (*F* (1, 46) = 4.74, *P* = 0.04, *η*^2^_partial_ = 0.10). Further analysis showed that, in the self-disclosure group, INS was significantly stronger in the negative condition than in the neutral condition (*P* < 0.001, Cohen’s *d* = 0.69). However, no significant tendency was observed in the non-disclosure group. Additionally, ΔINS (INS_Negative_ − INS_Neutral_) at CH6 was significantly larger in the self-disclosure group than in the non-disclosure group (*t* (46) = 3.49, *P* = 0.001, Cohen’s *d *= 1.01, 95% CI = [0.20, 0.76]; Figure 3A). Second, in the frequency 0.25–0.34 Hz, we found a significant interaction effect at CH1 (*F* (1, 46) = 12.29, *P* < 0.001, *η*^2^_partial_ = 0.21, 95% CI = [0.01, 0.68]). Note that this interaction remained borderline significant even after incorporating gender into the statistical model (*F* (1, 46) = 3.89, *P* = 0.055, *η*^2^_partial_ = 0.08). Further analysis showed that ΔINS at CH1 was significantly smaller in the self-disclosure group than in the non-disclosure group (*t* (46) = − 3.46, *P* = 0.001, Cohen’s *d* = 0.99, 95% CI = [−0.93, −0.24]; Figure 3A). CH6 and CH1 were roughly located at the left superior frontal gyrus and right middle frontal gyrus, respectively.

The distinct patterns observed in the two channels, operating at different frequency bands (CH6 at 0.09–0.10 Hz and CH1 at 0.25–0.34 Hz), were likely attributable to the affective and cognitive processes during sharing negative experience in the self-disclosure and non-disclosure groups. Consequently, we focused on ΔINS at the corresponding frequency bands of these two channels. We explored the relationships among ΔINS, ΔEmotional-empathy, ΔLiking, as well as ΔProsociality in the two disclosure groups. In the self-disclosure group, we found a significant correlation between ΔINS at CH6 and ΔLiking (*r* = 0.44, *P* = 0.03; Figure 3B), as well as a marginally positive correlation between ΔINS at CH6 and ΔProsociality (*r* = 0.35, *P* < 0.1). Accordingly, we conducted a mediation analysis for the self-disclosure group with ΔINS at CH6, ΔLiking and ΔProsociality as the independent, mediating and dependent variables, respectively. The analysis revealed a significant mediation effect (bootstrap ab = 2.37, 95% CI = [0.09, 6.15]; Figure 3C), suggesting that ΔLiking mediates between ΔINS at CH6 and ΔProsociality. In the non-disclosure group, however, ΔINS at CH1 did not correlate with ΔLiking (*r* = 0.14, *P* = 0.52) or ΔProsociality (*r* = − 0.11, *P* = 0.96).

## Discussion

In this study, we used fNIRS hyperscanning to examine the social outcomes of sharing negative experiences and the neural underpinnings. We observed that sharing negative (relative to neutral) experiences enhanced greater mutual prosociality and emotional empathy in the self-disclosure group compared to the non-disclosure group. In terms of brain activity, self-disclosure of negative (*vs* neutral) experiences induced stronger INS at the left superior frontal cortex (l-SFC). Importantly, mediation analyses further revealed that in the self-disclosure group, the increased emotional empathy and INS elicited by sharing negative experiences relative to sharing neutral experiences promoted the enhanced prosociality through increasing interpersonal liking.

Compared with sharing neutral experiences, sharing negative experiences elicited enhanced prosociality, emotional and cognitive empathy and liking. These social outcomes, as the foundations for social ties and connections, are crucial for well-being (Yoon *et al*., 2008; Liao and Weng, 2018) and physical health (Haslam *et al*., 2015; Hudson, 2017). During social communication, speaker and listener form a stable social connection that facilitates interpersonal cohesion, leading to both parties acting in a more harmonious way. Further, through sharing personal negative experiences, dyad members in our study accumulated interpersonal capital and felt closer to each other—greater harmony developed between the parties led to greater intention to behave prosocially (Eisenberg and Miller, 1987; Peters and Kashima, 2007). Feelings of social connection with others are facilitated by both behaviors (e.g. behavioral synchrony) and psychological processes (e.g. perceived partner responsiveness; Zhan, 2013). Past studies have consistently reported that positive affect increases the engagement of theory of mind for the person in need, in turn informing prosocial responses (i.e. feel good, do good; Fredrickson, 2003; Gaesser *et al*., 2017). Our observations extend these findings by showing that after sharing bad experiences, we are more inclined to act in a benevolent way.

Importantly, there was a larger valence condition effect (neutral *vs* negative) on prosociality when individuals shared their own experiences (i.e. self-disclosure) rather than those of a stranger (i.e. non-disclosure). Specifically, self-disclosure (*vs* non-disclosure) of negative experience seemed to evoke greater enhancement in prosocial behavior. This finding echoes prior observations that disclosing self-relevant information to a responsive partner bolsters feelings of closeness and intimacy, in turn promoting positive psychological and relational outcomes (Reis and Shaver, 1988; Regan *et al*., 2022). Our results also highlight the value of engaging in deep conversations, involving intimate self-disclosure. Disclosure in deep conversations was reported to be associated with enjoyment and feelings of closeness and may be seen as a rewarding and connecting experience (Kardas *et al*., 2022). Moreover, unacquainted dyads who engaged in a disclosure task (the Fast Friends procedure) subsequently reported more closeness and liking compared to those who engaged in small talk (Sprecher, 2021). These prior findings somewhat explain why we observed an enhanced valence condition effect of sharing experience on emotional empathy but not on cognitive empathy in the self-disclosure group compared to the non-disclosure group.

The INS increase elicited by sharing negative (compared to neutral) experiences was significant in the self-disclosure group at the l-SFC. Prior studies have uncovered that the frontal cortex critically contributes to emotion recognition and information encoding (Abrams *et al*., 2011), as well as self-other distinction (Ruby and Decety, 2004). In particular, the l-SFC was found to involve in personality and various cognitive domains, such as attention and response selection (Schilling *et al*., 2013). Thus, our findings suggest that l-SFC might be engaged in the establishing of a frame of emotional events. Moreover, previous studies have revealed that the synchronous brain activity across individuals underlying social interaction might reflect a sense of alignment, such as shared intention (Hu *et al*., 2017) or shared psychological perspectives (Lahnakoski *et al*., 2014). Consistent with previous studies (Tamir *et al*., 2015; Kulahci and Quinn, 2019; Pan *et al*., 2022), our findings of enhanced INS during negative experience sharing in the self-disclosure group might represent a high-level of cognitive alignment (e.g. joint attention and shared understanding toward shared narratives), essential to increasing interpersonal closeness between individuals.

Emotional empathy and INS both increased when individuals shared personal negative experiences. It was consistent with the previous findings that self-disclosure of emotional states may facilitate understanding of one’s own emotions (emotional empathy), which could promote social interaction and INS (Nummenmaa *et al*., 2012). It is important to highlight that the level of self-disclosure has been reported to correlate with the degree of interpersonal liking (Derlega *et al*., 1993), and INS has been linked to social factors such as interpersonal liking or closeness (Dikker *et al*., 2017). Our mediation analyses extended previous evidence and further revealed that increased emotional empathy and INS each affected prosociality through interpersonal liking. These findings imply that interpersonal attitude plays a crucial role in social behaviors.

Despite its strength, this study has several limitations. First, we followed a fixed sequence of the neutral condition first and then the negative condition. The purpose of this practice was to mitigate the impact of negative emotions on subsequent tasks. However, we cannot exclude the possibility that participants’ reported increase in prosocial intention over time (from negative to neutral) could be attributed to developing a better understanding of their partner throughout the interaction. Mitigating this concern, our empirical data in another experiment, as yet unpublished, showed that self-disclosure of negative experiences enhanced prosocial behavior and emotional empathy even when the task order was randomized. Second, in the current study, we implemented a narration phase prior to the communication phase. This practice ensured that all experiences were recounted in their entirety. However, inhibiting verbal responses from the listener during the narration phase may raise concerns about ecological validity, especially given its omission of the natural back-channeling that typically occurs in real-life conversations. Third, this study comprised 9 male–male dyads and 39 female–female dyads, introducing a potential gender imbalance that could influence prosocial behaviors and INS. It is worth noting that, even when gender was incorporated as covariate in the ANOVA, the overall results showed minimal alteration. Nevertheless, future research endeavors are encouraged to investigate this aspect.

In summary, our study demonstrates that self-disclosure of negative experiences promoted prosociality, emotional empathy and INS at the l-SFC between discloser and listener. Importantly, self-disclosure of negative experiences shapes prosociality via social dynamics (i.e. empathy and liking) and shared neural responses. In real-world scenarios, our research may inspire guidance on effectively expressing negative emotions, fostering mental and physical well-being and enhancing interpersonal interactions. Consequently, this study bears crucial implications for promoting prosocial behavior and fostering social harmony.

## Data Availability

The data and code demo are available in the Open Science Framework at https://osf.io/hmpe9/.
